# Supplementary material for: Dads at Mealtimes: Associations between Food Security, Household and Work Chaos, and Paternal Feeding Practices among Australian Fathers Living with Disadvantage
Source: Nutrients. 2024 Jan 8;16(2):205. doi: 10.3390/nu16020205 (PMC10820457; doi:10.3390/nu16020205)
Supplement: Supplementary file 1 [file nutrients-16-00205-s001.zip › nutrients-2797272-supplementary.pdf]

Supplementary Materials: Supplementary Tables S1-S5

Table S1. Item statistic for 18-item HFSSM reported in a sample of 222 participants.

**Response of HFSSM individual item (n = 222)**	**Affirmative responses** [a] N (%)	**Negatives responses** [a] N (%)
**Adult items**		
Worried food would run out	171 (77)	51 (23)
Food bought just didn't last	141 (64)	81 (37)
Couldn't afford to eat balanced meals	155 (70)	67 (30)
Adult cut size of meals or skipped meals	132 (60)	90 (41)
Adult ate less than felt they should	144 (65)	78 (35)
Adult cut size of meals or skipped meals in 3 or more months	102 (46)	120 (54)
Adult hungry buy didn't eat	118 (53)	104 (47)
Respondent lost weight	69 (31)	153 (69)
Adult did not eat for the whole day	43 (19)	179 (81)
Adult did not eat for whole day in 3 or more months	29 (13)	193 (87)
**Child items**		
Relied on few kinds of low-cost food for children	156 (70)	66 (30)
Couldn't feed the children a balanced meal	108 (49)	114 (51)
Children were not eating enough	35 (16)	187 (84)
Cut size of children's meals	9 (4)	213 (96)
Children were hungry	12 (5)	210 (95)
Children skipped meals	5 (2)	217 (98)
Children skipped meals in 3 or more months	4 (2)	218 (98)
Children did not eat for whole day	6 (3)	216 (97)

[a] Affirmative responses were 'sometimes' or 'often true'; 'yes' or 'almost every month or some months'; negative responses were 'never true'; 'no' or 'only 1 or 2 months'.

Table S2. Food security status assessed by HFSSM in binary and categorical form, and NHS single item in a sample of 222 participants, and separately for young and older child groups.

	**Total (n =222)**	**Younger child group [<2 years] (n =88)**	**Older child group [2 - 5 years] (n = 134)**
**Australian NHS-1 item, n (%)**			
Food secure	141 (64)	61 (58)	80 (50)
Food insecure	81 (37)	27 (26)	54 (34)
**HFSSM, n (%)**			
Food secure	52 (23)	21 (24)	31 (23)
- High food security	22 (10)	9 (9)	13 (8)
- Marginal food security	30 (14)	12 (11)	18 (11)
Food insecure	170 (77)	67 (76)	103 (77)
- Low food security	72 (32)	30 (29)	42 (26)
- Very low food security	98 (44)	37 (35)	61 (38)

NHS, National Health Survey; HFSSM, Household Food Security Survey Module.

Table S3. Means, standard deviation and internal reliability of feeding practices for the separate younger and older child groups.

	M±SD	Cronbach's α	Missing data (%)
**Younger child group [<2 years] (n =105)**<br>- **FPSQ-S**			
Feeding on demand [a, d] (4 items)	3.5±0.6	0.55	11 (11)
Foods to calm [b] (6 items)	2.3±0.8	0.88	12 (11)
Persuasive feeding [b] (7 items)	2.8±0.9	0.86	11 (11)
Parent-led feeding [b] (4 items)	2.3±0.8	0.75	11 (11)
Family meal environment [a] (4 items)	3.9±0.9	0.75	53[e] (51)
Using(non-) food rewards [b] (9 items)	1.9±0.9	0.94	55[e] (52)
**Older child group [2 -5 years] (n =159)**			
- **FPSQ-28**			
Reward for behaviour [b] (4 items)	2.7±0.8	0.78	14 (9)
Reward for eating [b] (4 items)	2.5±0.9	0.86	14 (9)
Persuasive feeding [b] (6 items)	3.4±0.6	0.70	14 (9)
Covert restriction [a] (4 items)	2.8±0.9	0.82	15 (9)
Structured meal setting [a] (3 items)	3.7±0.9	0.81	15 (9)
Structured meal timing [a] (3 items)	3.4±0.6	0.39	15 (9)
Overt restriction [b] (4 items)	3.7±0.9	0.68	23 (15)
- **FPI**			
Offer new foods [c] (3 items)	3.7±0.6	0.83	24 (15)
Encourage exploration of new foods [c] (3 items)	3.9±0.9	0.90	24 (15)
Urge child to eat new foods [c] (4 items)	3.6±0.6	0.58	25 (16)
Repeated presentation of new foods [c] (3 items)	3.4±0.8	0.69	25 (16)

M±SD, Mean ± standard deviation; FPSQ-S/28, Feeding Practices and Structure Questionnaire; FPI, Food Parenting Inventory

[a] Construct related to structure

[b] Construct related to coercive control

[c] Construct related to autonomy support

[d] Higher feeding on demand score indicated more adherence to feeding routine.

[e] This was due to these constructs applying to fathers with children aged one year or above (n =56). True missing values were four to six (7-11%) for these constructs.

Table S4. Count and proportion for family meal setting, and bivariate analyses with food security status, household and work chaos for older child group.

**Older child group [2 – 5 years]**		**Total count** n=134	**HFSSM food security** [a]		**CHAOS score** [b] n =133	**Work chaos score** [b] n =133
			**Food secure** n=31	**Food insecure** n=103		
		n (%)			M±SD	
**Family meal setting**	Never	5 (4)	0	5 (5)	17.80±3.90	1.20±1.30
	Rarely	9 (7)	2 (7)	7 (7)	18.56±4.25	1.56±1.74
	Sometimes	23 (17)	6 (19)	17 (17)	16.26±3.97	1.04±1.07
	Often	54 (40)	11 (36)	43 (42)	16.76±4.43	1.06±1.27
	Always	43 (32)	12 (39)	31 (30)	15.14±4.77	1.48±1.33
			p = 0.755		p =0.183	p =0.471

HFSSM, Household Food Security Survey Module; CHAOS, Confusion, Hubbub, and Order Scale

[a] Fisher's Exact test was used to test for household food security status

[b] One-way ANOVA was used to test for CHAOS and work chaos scores

Table S5. Regression models examining individual effects and combining effects of food security, household and work chaos on paternal feeding practices for younger and older child groups.

			Regression Models									
			1		2		3		4		5	
**Feeding practices**		**IV**	**B (95% CI)**	**p-value**	**B (95% CI)**	**p-value**	**B (95% CI)**	**p-value**	**B (95% CI)**	**p-value**	**B (95% CI)**	**p-value**
**Younger child group [<2 years]** [a]												
Coercive control	Using food to calm	FI [b]	0.291 (-0.109, 0.691)	0.152	-	-	-	-	0.208 (-0.175, 0.592)	0.284	0.213 (-0.174, 0.601)	0.277
		HC	-	-	0.067 (0.003, 0.104)	<0.001	-	-	0.066 (0.029, 0.103)	<0.001	0.069 (0.031, 0.108)	<0.001
		WC	-	-	-	-	-0.057 (-0.193, 0.078)	0.404	-	-	-0.086 (-0.018, 0.073)	0.403
			R^2: 0.013 (n=87)		R^2: 0.124*** (n=86)		R^2: -0.004 (n= 84)		R^2: 0.126*** (n=86)		R^2: 0.132** (n=84)	
	Persuasive feeding	FI [b]	0.530 (0.091, 0.969)	0.019	-	-	-	-	0.514 (0.083, 0.944)	0.02	0.511 (0.072, 0.95)	0.023
		HC	-	-	0.065 (0.022, 0.107)	0.003	-	-	0.062 (0.02, 0.103)	0.004	0.063 (0.02, 0.106)	0.005
		WC	-	-	-	-	-0.054 (-0.206, 0.099)	0.487	-	-	-0.049 (-0.191, 0.094)	0.498
			R^2: 0.052* (n=88)		R^2: 0.087** (n=87)		R^2: -0.006 (n=85)		R^2: 0.134*** (n=87)		R^2: 0.126** (n=85)	
	Parent-led feeding	FI [b]	0.275 (-0.128, 0.678)	0.178	-	-	-	-	0.296 (-0.117, 0.708)	0.157	0.313 (-0.103, 0.729)	0.139
		HC	-	-	0.021	0.293	-	-	0.019	0.332	0.021	0.317

					(-0.019, 0.061)				(-0.02, 0.059)		(0.02, 0.061)	
		WC	-	-	-	-	-0.007 (-0.143, 0.129)	0.919	-	-	-0.004 (-0.139, 0.131)	0.952
			R^2: 0.01(n=88)		R^2: 0.001(n=87)		R^2: -0.012 (n=86)		R^2: 0.013 (n=87)		R^2: 0.005 (n=86)	
Structure	Family meal environment	FI [b]	-0.025 (-0.59, 0.539)	0.928	-	-	-	-	0.002 (-0.558, 0.562)	0.994	0.016 (-0.555, 0.587)	0.955
		HC	-	-	-0.039 (-0.094, 0.015)	0.153	-	-	-0.039 (-0.094, 0.016)	0.158	-0.036 (-0.094, 0.022)	0.218
		WC	-	-	-	-	-0.052 (-0.231, 0.127)	0.561	-	-	-0.053 (-0.233, 0.127)	0.555
			R^2: -0.021 (n=49)		R^2: 0.023 (n=49)		R^2: -0.014 (n=48)		R^2: 0.001 (n=49)		R^2: -0.024(n=48)	
**Older child group [2 – 5 years] [a]**												
Coercive control	Reward for behaviour	FI [b]	0.328 (-0.003, 0.660)	0.021	-	-	-	-	0.178 (-0.156, 0.512)	0.294	0.168 (-0.166, 0.501)	0.322
		HC	-	-	0.046 (0.016, 0.077)	0.003	-	-	0.042 (0.011, 0.074)	0.008	0.039 (0.008, 0.071)	0.015
		WC	-	-	-	-	0.096 (-0.012, 0.204)	0.082	-	-	0.07 (-0.037, 0.177)	0.2
			R^2: 0.021 (n=134)		R^2: 0.057** (n=133)		R^2: 0.015 (n=133)		R^2: 0.058** (n=133)		R^2: 0.062** (n=133)	
	Reward for eating	FI [b]	0.145 (-0.236, 0.525)	0.453	-	-	-	-	0.034 (-0.358, 0.426)	0.865	0.032 (-0.036, 0.426)	0.871
		HC	-	-	0.037 (0.002, 0.073)	0.041	-	-	0.038 (0.000, 0.073)	0.051	0.036 (-0.001, 0.073)	0.057
		WC	-	-	-	-	0.030 (-0.096, 0.156)	0.637	-	-	0.009 (-0.0117, 0.136)	0.883

			R^2: -0.003 (n=134)		R^2: 0.024* (n=133)		R^2: -0.006 (n=133)		R^2: 0.017 (n=133)		R^2: 0.009 (n=133)	
	Persuasive feeding	FI [b]	0.260 (0.015, 0.505)	0.038	-	-	-	-	0.245 (-0.011, 0.501)	0.06	0.240 (-0.016, 0.497)	0.066
		HC	-	-	0.014 (-0.01, 0.038)	0.244	-	-	0.009 (-0.015, 0.033)	0.469	0.007 (-0.017, 0.032)	0.545
		WC	-	-	-	-	0.042 (-0.04, 0.124)	0.31	-	-	0.032 (-0.031, 0.114)	0.445
			R^2: 0.025* (n=134)		R^2: 0.003 (n=133)		R^2: 0.000 (n=133)		R^2: 0.022 (n=133)		R^2: 0.019 (n=133)	
	Overt restriction	FI [b]	0.043 (-0.312, 0.399)	0.810	-	-	-	-	-0.121 (-0.478, 0.235)	0.503	-0.129 (-0.486, 0.228)	0.477
		HC	-	-	0.036 (0.004, 0.069)	0.029	-	-	0.039 (0.005, 0.072)	0.023	0.037 (0.003, 0.070)	0.033
		WC	-	-	-	-	0.067 (-0.047, 0.181)	0.249	-	-	0.05 (-0.065, 0.165)	0.389
			R^2: -0.007 (n=134)		R^2: 0.029* (n=133)		R^2: 0.003 (n=133)		R^2: 0.025 (n=133)		R^2: 0.023 (n=133)	
Structure	Covert restriction	FI [b]	-0.013 (-0.376, 0.349)	0.942	-	-	-	-	0.094 (-0.279, 0.466)	0.62	0.095 (-0.279, 0.470)	0.615
		HC	-	-	-0.02 (-0.054, 0.014)	0.256	-	-	-0.022 (-0.056, 0.013)	0.224	-0.021 (-0056, 0.014)	0.242
		WC	-	-	-	-	-0.022 (-0.014, 0.097)	0.719	-	-	-0.013 (-0.133, 0.108)	0.837
			R^2: -0.008 (n=134)		R^2: 0.002 (n=133)		R^2: -0.007* (n=133)		R^2: -0.003 (n=133)		R^2: -0.011 (n=133)	
	Structured meal setting	FI [b]	-0.210 (-0.558, 0.139)	0.236	-	-	-	-	-0.123 (-0.484, 0.237)	0.5	-0.123 (-0.486, 0.239)	0.502
		HC	-	-	-0.023 (-0.056,	0.165	-	-	-0.021 (-0.054,	0.23	-0.021 (-0.055,	0.236

					0.01)				0.013)		0.014)	
		WC	-	-	-	-	-0.014 (-0.129, 0.101)	0.815	-	-	0.001 (-0.116, 0.117)	0.987
			R^2: 0.003 (n=134)		R^2: 0.007 (n=133)		R^2: -0.007 (n=133)		R^2: 0.003 (n=133)		R^2: -0.005 (n=133)	
Autonomy support	Offer new foods	FI [b]	-0.011 (-0.366, 0.147)	0.400	-	-	-	-	-0.052 (-0.316, 0.212)	0.699	-0.045 (-0.310, 0.219)	0.735
		HC	-	-	-0.027 (-0.051, -0.003)	0.025	-	-	-0.026 (-0.051, -0.002)	0.037	-0.025 (-0.050, 0.000)	0.054
		WC	-	-	-	-	-0.058 (-0.142, -0.027)	0.178	-	-	-0.043 (-0.128, 0.042)	0.317
			R^2: -0.002 (n=134)		R^2: 0.03 (n=133)		R^2: 0.006 (n=133)		R^2: 0.024 (n=133)		R^2: 0.024 (n=133)	
	Exploration of new foods	FI [b]	-0.131 (-0.485, 0.224)	0.467	-	-	-	-	-0.088 (-0.458, 0.282)	0.638	-0.083 (-0.454, 0.288)	0.659
		HC	-	-	-0.019 (-0.053, 0.015)	0.269	-	-	-0.017 (-0.052, 0.018)	0.332	-0.016 (-0.051, 0.02)	0.382
		WC	-	-	-	-	-0.046 (-0.163, 0.072)	0.442	-	-	-0.035 (-0.154, 0.084_	0.563
			R^2: -0.004 (n=134)		R^2: 0.002 (n=133)		R^2: -0.003 (n=133)		R^2: -0.004 (n=133)		R^2: -0.009 (n=133)	
	Repeated presentation of new foods	FI [b]	-0.194 (-0.505, 0116)	0.217	-	-	-	-	-0.074 (-0.389, 0.241)	0.642	-0.078 (-0.394, 0.239)	0.629
		HC	-	-	-0.041 (-0.07, -0.012)	0.005	-	-	-0.04 (-0.069, -0.01)	0.009	-0.04 (-0.07, -0.011)	0.008
		WC	-	-	-	-	-0.002 (-0.105, 0.101)	0.972	-	-	0.022 (-0.079, 0.124)	0.664
			R^2: 0.004 (n=134)		R^2: 0.051^{**} (n=133)		R^2: -0.008 (n=133)		R^2: 0.045^{*} (n=133)		R^2: 0.039^{*} (n=133)	

B, Unstandardised coefficients; CI, Confidence Interval; FI, Food insecurity; HC, Household chaos; IV, Independent variable; R^2, Coefficient of determination; WC, Work chaos

[a] Younger child group completed FPSQ-S; Older child group completed FPSQ-28 and FPI

Coding for categorical variables (where 0 is the reference group):

[b] Dummy coded: food insecure = 1, food secure = 0

* = $p<0.05$, ** = $p<0.01$, *** = $p<0.001$
